# What Are Reasons for the Large Gender Differences in the Lethality of Suicidal Acts? An Epidemiological Analysis in Four European Countries

**DOI:** 10.1371/journal.pone.0129062

**Published:** 2015-07-06

**Authors:** Roland Mergl, Nicole Koburger, Katherina Heinrichs, András Székely, Mónika Ditta Tóth, James Coyne, Sónia Quintão, Ella Arensman, Claire Coffey, Margaret Maxwell, Airi Värnik, Chantal van Audenhove, David McDaid, Marco Sarchiapone, Armin Schmidtke, Axel Genz, Ricardo Gusmão, Ulrich Hegerl

**Affiliations:** 1 Department of Psychiatry and Psychotherapy, University of Leipzig, Leipzig, Saxonia, Germany; 2 Institute of Behavioral Sciences, Semmelweis University Budapest, Budapest, Hungary; 3 Department of Health Psychology, University Medical Center, Groningen, the Netherlands; 4 CEDOC, Department of Psychiatry and Mental Health, Universidade Nova de Lisboa, Lisbon, Portugal; 5 National Suicide Research Foundation and Department of Epidemiology and Public Health, University College Cork, Cork, Ireland; 6 Nursing, Midwifery and Allied Health Professions Research Unit, University of Stirling, Stirling, Scotland, United Kingdom; 7 Estonian-Swedish Mental Health and Suicidology Institute and Tallinn University, Tallinn, Estonia; 8 LUCAS Center for care research and consultancy at Katholieke Universiteit Leuven, University of Leuven, Leuven, Belgium; 9 London School of Economics, London, United Kingdom; 10 Department of Medicine and Health Sciences, University of Molise, Campobasso, Italy, National Institute for Health, Migration and Poverty, Rome, Italy & University “Gabriele d’Annunzio” Foundation, Chieti, Italy; 11 Department of Psychiatry, Psychosomatics and Psychotherapy, Universität Würzburg, Würzburg, Germany; 12 Department of Psychiatry, Psychotherapy and Psychosomatic Medicine, Otto-von-Guericke University Magdeburg, Magdeburg, Germany; 13 Department of Psychiatry and Mental Health, CEDOC, Universidade Nova de Lisboa & ISPUP, Institute of Public Health, University of Porto, Porto, Portugal; Medical University of Vienna, AUSTRIA

## Abstract

**Background:**

In Europe, men have lower rates of attempted suicide compared to women and at the same time a higher rate of completed suicides, indicating major gender differences in lethality of suicidal behaviour. The aim of this study was to analyse the extent to which these gender differences in lethality can be explained by factors such as choice of more lethal methods or lethality differences within the same suicide method or age. In addition, we explored gender differences in the intentionality of suicide attempts.

**Methods and Findings:**

**Methods**. Design: Epidemiological study using a combination of self-report and official data. Setting: Mental health care services in four European countries: Germany, Hungary, Ireland, and Portugal. Data basis: Completed suicides derived from official statistics for each country (767 acts, 74.4% male) and assessed suicide attempts excluding habitual intentional self-harm (8,175 acts, 43.2% male).

**Main Outcome Measures and Data Analysis.** We collected data on suicidal acts in eight regions of four European countries participating in the EU-funded “OSPI-Europe”-project (www.ospi-europe.com). We calculated method-specific lethality using the number of completed suicides per method * 100 / (number of completed suicides per method + number of attempted suicides per method). We tested gender differences in the distribution of suicidal acts for significance by using the χ^2^-test for two-by-two tables. We assessed the effect sizes with phi coefficients (φ). We identified predictors of lethality with a binary logistic regression analysis. Poisson regression analysis examined the contribution of choice of methods and method-specific lethality to gender differences in the lethality of suicidal acts.

**Findings Main Results:**

Suicidal acts (fatal and non-fatal) were 3.4 times more lethal in men than in women (lethality 13.91% (regarding 4106 suicidal acts) versus 4.05% (regarding 4836 suicidal acts)), the difference being significant for the methods hanging, jumping, moving objects, sharp objects and poisoning by substances other than drugs. Median age at time of suicidal behaviour (35–44 years) did not differ between males and females. The overall gender difference in lethality of suicidal behaviour was explained by males choosing more lethal suicide methods (odds ratio (OR) = 2.03; 95% CI = 1.65 to 2.50; p < 0.000001) and additionally, but to a lesser degree, by a higher lethality of suicidal acts for males even within the same method (OR = 1.64; 95% CI = 1.32 to 2.02; p = 0.000005). Results of a regression analysis revealed neither age nor country differences were significant predictors for gender differences in the lethality of suicidal acts. The proportion of serious suicide attempts among all non-fatal suicidal acts with known intentionality (NFSAi) was significantly higher in men (57.1%; 1,207 of 2,115 NFSAi) than in women (48.6%; 1,508 of 3,100 NFSAi) (χ^2^ = 35.74; p < 0.000001).

**Main limitations of the study:**

Due to restrictive data security regulations to ensure anonymity in Ireland, specific ages could not be provided because of the relatively low absolute numbers of suicide in the Irish intervention and control region. Therefore, analyses of the interaction between gender and age could only be conducted for three of the four countries. Attempted suicides were assessed for patients presenting to emergency departments or treated in hospitals. An unknown rate of attempted suicides remained undetected. This may have caused an overestimation of the lethality of certain methods. Moreover, the detection of attempted suicides and the registration of completed suicides might have differed across the four countries. Some suicides might be hidden and misclassified as undetermined deaths.

**Conclusions:**

Men more often used highly lethal methods in suicidal behaviour, but there was also a higher method-specific lethality which together explained the large gender differences in the lethality of suicidal acts. Gender differences in the lethality of suicidal acts were fairly consistent across all four European countries examined. Males and females did not differ in age at time of suicidal behaviour. Suicide attempts by males were rated as being more serious independent of the method used, with the exceptions of attempted hanging, suggesting gender differences in intentionality associated with suicidal behaviour. These findings contribute to understanding of the spectrum of reasons for gender differences in the lethality of suicidal behaviour and should inform the development of gender specific strategies for suicide prevention.

## Introduction

Suicidal acts—completed and attempted suicides—are significant public health problems worldwide: the World Health Organization (WHO) reports around 804,000 suicides in the year 2012 [1]. The number of attempted suicides is estimated to be more than 20 times higher [1]. Men have a greater risk for completed suicides. The male-female rate ratio of suicide in the WHO European region is 4: 1, the highest in the world [2]. This gender difference in suicide rates exists despite men engaging less frequently in suicide attempts [3,4]. A lower rate of attempted and a higher rate of completed suicides result in major gender differences in the lethality of suicidal behaviour. Lethality has been found to be 4.78 times higher in males than in females [5]. At first sight this seems to stem from the fact that males more often use high-risk methods such as hanging, jumping, use of fire arms, being run over and drowning than females [3,4,6–8]. In contrast, intoxications, which are survived by more than 90% of individuals are by far the most frequently used method by females (87.5% of all female suicidal acts, but only 38.6% of male suicidal acts represent intoxications) [3]. However, even within the same method the outcome has been found to be more lethal for males [3]. In fact, other factors may contribute to these gender differences and are discussed in the literature. Some of them are empirically proven, others have not been sufficiently studied yet and are listed for theoretical reasons. These factors can be categorized into three groups:

1. Factors which influence lethality of a certain suicidal act largely independently from the choice of more or less lethal suicidal methods. Among these is the social and communicative context of the suicidal act. Men's reluctance to seek help, to communicate their acute crisis and their higher social isolation might reduce the chance of getting help in time [3,9,10], e.g. after an acute intoxication. Furthermore, the additional involvement of alcohol or drug consumption is more often found in males than in females [11] and is likely to increase the lethality of suicide attempts, especially in patients with mood disorders [12]. Generally, a combination of suicide methods is more common in males than in females [13,14].
2. Factors which influence lethality via the choice of a more lethal suicide method. Among these are social acceptability, model learning (cognitive availability) and ease of access to or technical ability in the chosen suicide method [6]. Knowledge of and access to firearms can be expected to be easier for males than females. The technical skills required for hanging might also prevent females from choosing this highly lethal method [3,15]. Furthermore, it is proposed that women may be more concerned about their appearance and try to ensure that their body and face are not severely injured [4,15], which might also be a cause for their preference for self-poisoning and drowning [6].
3. Factors which influence lethality via both choice of a more lethal suicidal method and a more lethal outcome within the same method. Higher age is associated with a more lethal outcome of suicidal acts in general and may also influence the choice of the suicidal method. Males may also differ from females in terms of the decisiveness to die, resulting in a strong intentionality of suicidal acts. This may explain the choice of more lethal suicidal methods, as well as a higher lethality in general. There may be also gender differences in the dynamic of the suicidal process (e.g. higher impulsivity of the suicidal acts in males).

**Fig 1:**
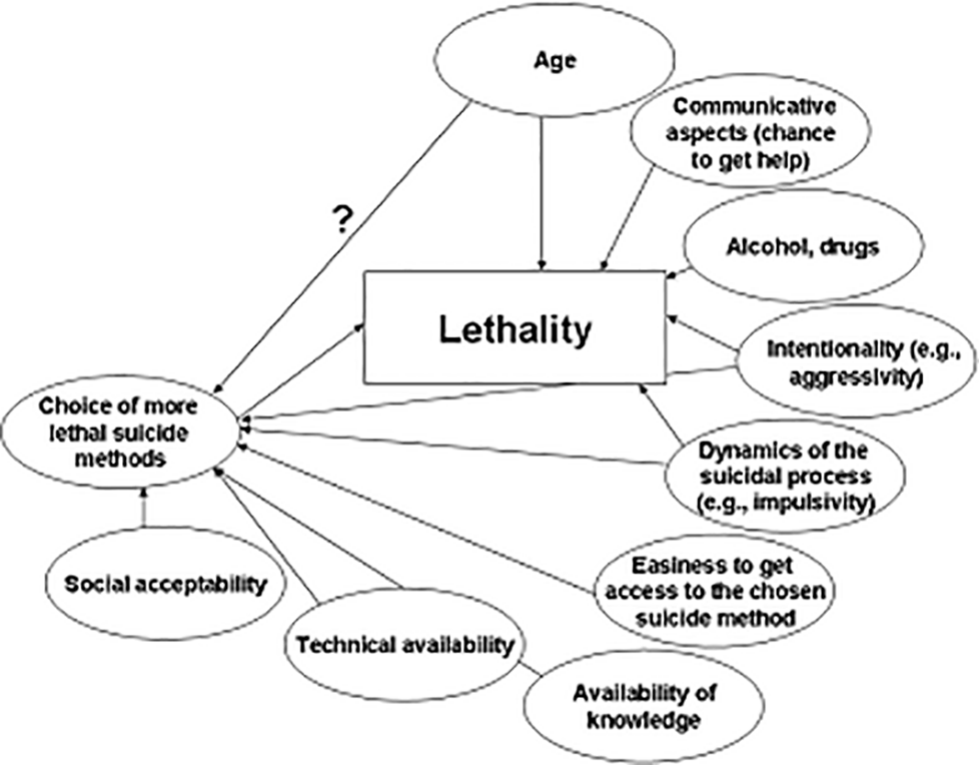
Potential impact factors on gender differences in lethality of suicidal acts.

This list of factors is likely to be far from complete and the interrelatedness among the different factors contributes to the complexity of the picture. To improve preventive actions and gender-specific care for suicidal patients, it is important to gain a better understanding of the relevance of the many factors associated with the large gender differences in lethality of suicidal acts. Fig 1 presents a simplified model of the possible impact of a number of empirically proven and other potential factors on gender differences in lethality of suicidal acts, influencing them either directly or via the choice of a more or less lethal method.

Analysis of gender differences in lethality of suicidal behaviour requires data on attempted suicides which are not collected in national registers, with the exception of Ireland. One of the few studies intensively dealing with the assessment of data on attempted suicides was the “WHO/EURO Multicentre Study on Suicidal Behaviour” conducted from 1989–1992 [8,16]. Other studies including attempted suicides and the methods used mostly refer to one or two regions in Europe [17]. Ajdacic-Gross et al. [6] undertook international comparisons of suicide methods, but did not refer to attempted suicides or lethality. Cibis et al. [3] recently analysed the gender-specific lethality of suicide methods in two regions in Germany and concluded that the same suicide method tends to be more lethal when carried out by men. This effect remained significant after controlling for age, which was higher for men than for women conducting a suicidal act [3].

The aim of this international prospective cohort study was to enhance our understanding of the gender differences in lethality of suicidal acts in an international context and to replicate and extend previous findings [3]. More specifically the study will address 1) the extent to which lethality differences are explained by gender differences in the choice of more or less lethal suicidal methods, 2) gender differences in lethality within certain suicidal methods, 3) the interaction of the two factors in predicting the extend of gender differences in lethality and 4) whether gender differences in the lethality of suicidal acts differ between countries and age groups. In addition, lethality differences in age and gender differences in the intentionality of suicide attempts were explored.

## Materials and Methods

### OSPI-Europe

The present study was conducted within the context of an international prospective cohort study. The data presented here were collected in Germany, Hungary, Ireland, and Portugal within the EU-funded project OSPI-Europe (“Optimising suicide prevention programmes and their implementation in Europe”, 7^th^ Framework Programme). A multilevel community based suicide prevention programme was implemented in one region per country, with a second region per country acting as a control region. This programme is based on the 4-level intervention approach developed within the previous EAAD-project (European Alliance Against Depression) [18,19]. Suicidal acts (completed and attempted suicides) were assessed as an outcome parameter in all intervention and control regions. Intervention effects will be reported elsewhere. The intervention was conducted for at least 1.5 years after a one-year baseline. The exact time spans for the intervention activities were as follows: June 2009 –March 2011 (Germany); January 2010 –June 2011 (Hungary), April 2010 –September 2011 (Portugal and Ireland).

### Ethics Statement

The OSPI-Europe research project was executed in accordance with the principles laid down in the Helsinki declaration (2000). The OSPI interventions took place in Germany, Hungary, Ireland and Portugal. Each country had an intervention and a comparison/control site. Each of the four research teams sought ethical review and gained approval from the relevant bodies in each country: Ethics Commission of the Medical Faculty, University of Leipzig, Germany (refs. 248–2007 and 140-2009-06072009); Semmelweis University Regional and Institutional Committee of Science and Research Ethics, Hungary (ref. TUKEB 149/2009), Ethics Research Committee of the Mid-West Regional Hospital, Limerick City and County, Ireland (no reference number, letter of approval dated 25/06/2009) and Clinical Research Ethics Committee, Merlin Park University Hospital, Galway City and County, Ireland (ref. C.A. 271); and the Ethical Committee of the Faculty of Medical Sciences, New University of Lisbon, Portugal (ref. CE/DP/7-2009). For the assessment of suicide attempts through patient records (Hungary, Portugal, partly Germany) or a routine procedure (Ireland) neither written nor verbal consent of patients was obtained. In case of interview participation, written informed consent was not obtained in order to not overwhelm the patients after suicide attempt with information and documentation. Informed verbal consent was obtained at the beginning of the interview by trained staff. A filled-out interview protocol functioned as documentation of participant consent. The ethics committee of each of the participating intervention regions approved this procedure prior to initiating the study.

### Suicidal acts

The term “suicidal acts” represents the sum of completed suicides and attempted suicides. Data on suicidal acts were available for two participating regions in each country for three years for Germany (June 2008 to May 2011), Hungary (January 2008 to December 2010), Ireland (April 2009 to March 2012) and Portugal (April 2009 to March 2012).

### Assessment of completed suicides

The definition of completed suicides followed the ICD-10 codes (International Statistical Classification of Diseases, tenth revision) [20] for intentional self harm as an external cause of morbidity and mortality (X60-X84). Data on completed suicides were obtained from official statistics offices in the four countries for both the intervention and control regions. For each case the method indicated on the death certificate was assessed together with age and sex. Due to restrictive data security regulations in Ireland, specific ages could not be provided because of the relatively low absolute numbers of suicide in the intervention and control region. Therefore analyses of the interaction between gender and age could only be conducted for three of the four countries.

### Assessment of attempted suicides

An attempted suicide was defined as “an act with non-fatal outcome, in which an individual deliberately initiates a non-habitual behaviour that, without intervention from others, will cause self-harm, or deliberately ingests a substance in excess of the prescribed or generally recognised therapeutic dosage, and which is aimed at realizing changes which the subject desired via the actual or expected physical consequences” [21]. This includes suicidal behaviour elsewhere referred to as parasuicide (including parasuicidal pause and parasuicidal gesture) [22]. Habitual intentional self harm, which is usually related to emotional regulation, was excluded by this definition.

Data on attempted suicides were obtained by the four participating countries for the two regions each following the above named standard definition and a given set of variables. A standardised questionnaire for data assessment and a codebook listing the variables for the registration of attempted suicides was used by all partners to ensure comparability in data obtainment. Coverage of data assessment (number of participating centres) and exact method depended on the local circumstances as described in the following. It was assured that data assessment was consistent over time within each centre.

In Hungary and Portugal, all admissions to hospitals due to attempted suicide were assessed via retrospective data collection using patient records. In Germany, the data assessment procedure followed a prospective design assessing data via personal interviews by trained staff. Staff changes in two of four participating centres may have led to irregularities in data assessment, therefore interview data in those two centres were replaced by data stemming from a retrospective assessment of patient records. For the other two German centres it could be verified that the data assessment procedure was applied consistently over time. In Ireland, data on cases of attempted suicides presented to a hospital are routinely collected by trained data registration officers using standard methods of case ascertainment and definition, processed by the National Registry of Deliberate Self Harm [23], from which the required data could be retrieved.

Unclear cases which could not instantly be categorised as a suicidal act or any other behaviour, for example cases to represent habitual self harm, were pooled and blinded for timespan and region and then categorised by internal experts.

Up to five different methods were assessed per suicide attempt. When several methods were used, the most lethal one according to previous results [3,24] was recorded as the primary method and forms the basis for the present analyses.

### Methods used for suicidal acts

Methods of suicidal acts were assessed according to ICD-10 codes X60-X84 [20] and categorised in nine groups: poisoning by drugs (X60-X64), poisoning by other means (X65-X69), hanging (X70), drowning (X71), firearms (X72-X75), sharp objects (X78), jumping (X80), moving objects (X81,X82), other methods (X76, X77, X79, X83, X84).

### Definition of lethality, high / low risk methods and intentionality of suicidal acts

Lethality was calculated as the ratio of completed suicides to suicidal acts: lethality = number of completed suicides * 100 / number of suicidal acts. A cut-off point between high-risk methods and low-risk methods was set at the median (10.8%), so that high-risk methods show a lethality of 10.8% or above, whereas low-risk methods show a lethality below 10.8%.

Based on the categorical Feuerlein scale [22] which had been developed in order to classify different psychological motives for suicidal acts and implies three types of them (parasuicide pauses, parasuicide gestures, serious suicide attempts), the intentionality of suicide attempts was assessed by using the following item:

“Classification of suicide attempt:

01 –deliberate self-harm (non-habitual)
02 –parasuicide pause/temporary rest
03 –parasuicide gesture
04 –serious suicide attempt
09 –not known”

In this context, the term “parasuicide pause” refers to the wish to interrupt a situation which is considered to be not sufferable. Regarding the parasuicide gesture, the suicide attempt has a primary appeal function without suicidal intent. For serious suicide attempts, the primary motive is the intention to die. Non-habitual deliberate self-harm represents a residual category of suicidal acts which is characterized by the primary intention to injure oneself and does not include habitual deliberate self-harm (e.g. of persons with a borderline personality disorder cutting themselves).

### Statistical analysis

Gender differences in the lethality of suicidal acts were tested for significance by using the ^χ2^-test for two-by-two tables. The effect sizes were assessed with phi coefficients (φ). These analyses were conducted for the total sample and afterwards for the national samples separately. Χ^2^-tests were also applied for the analysis of gender differences in the distribution of different types of suicide attempts according to the Feuerlein scale.

Moreover, gender differences for completed suicides were computed for the four possible different constellations regarding the presence (1 = yes; 0 = no) of gender differences for

- the choice of suicide methods;
- method-specific lethality of suicidal acts.

Gender-specific frequencies of completed suicides for these 2^2^ constellations (1–1, 1–0, 0–1, 0–0) and the resulting lethalities of suicidal acts were compiled in one data file. In a subsequent step, a Poisson regression analysis was applied in order to investigate whether gender differences in the variables a-b were significantly associated with gender differences regarding the lethality of suicidal acts. The suicide frequencies were the dependent variable in a generalized linear model based on a Poisson distribution, with the offset term being the natural logarithm of the number of suicidal acts. Thus, estimated marginal means for the lethality of suicidal acts could be computed. The model included the main and all possible interaction effects of the three predictors: gender; presence of gender differences for the choice of suicide methods (a); and presence of gender differences for method-specific lethality of suicidal acts (b). In this context, positive odds ratios for the interaction terms gender x a and gender x b reflect significant contributions of gender differences in the choice of methods and method-specific lethality of suicidal acts to gender differences in the lethality of suicidal acts in general.

Further significant predictors of lethality differences were identified by a binary logistic regression analysis. The binary dependent variable was lethality (1 = completed suicide; 0 = attempted suicide). The independent variables were as follows: gender (primary variable of interest; male, female; reference category: female), country (Germany, Hungary, Ireland, Portugal; reference category: Germany), suicide methods (with 9 categories (see above); reference category: poisoning by drugs, because this method is known to have a low lethality), the interaction of the factors gender and method of suicidal acts as well as gender and country. The listed interactions are of special interest because they allow the identification of relevant predictors for gender differences in the lethality of suicidal acts. For quantifying the strength of associations between these variables and lethality odds ratios and the corresponding 95% confidence intervals (CI) were applied. A similar binary logistic regression analysis was conducted with age as additional independent variable. This analysis was restricted to Germany, Hungary, and Portugal since information about age was not available for suicides in Ireland.

These statistical tests were performed using the statistical software package for IBM (International Business Machines Corporation) SPSS (Statistical Package for Social Sciences) Statistics 20 for Windows (International Business Machines Corporation (IBM), New York, USA). The significance level was set at 0.05.

## Results

The OSPI-Europe study regions, timespans of data assessment, population numbers, and numbers of assessed suicidal acts were described in Table 1.

**Table 1:** OSPI- Europe regions, timespans of data assessment, population numbers and numbers of assessed suicidal acts.

Country	Germany		Hungary		Ireland		Portugal	
Timespan	Jun 2008 –May 2011		Jan 2008 –Dec 2010		Apr 2009 –Mar 2011		Apr 2009 –Mar 2011	
**City**	**Leipzig**	**Magdeburg**	**Miskolc**	**Szeged**	**Limerick**	**Galway**	**Amadora**	**Almada**
**Population**	513,000	230,100	170,300	169,000	190,600	241,300	170,900	166,000
**– Male**	248,700	111,200	78,600	77,400	95,700	120,000	81,400	80,100
**- Female**	264,300	118,900	91,700	91,600	94,900	121,300	89,500	85,900
**Suicidal Acts**	1407	569	763	578	2267	1895	743	720
**– Male**	659	261	405	271	1106	927	258	219
**- Female**	748	308	358	307	1161	968	485	501
**Completed Suicides**	212	60	141	126	65	72	42	49
**– Male**	148	43	114	85	57	55	34	35
**- Female**	64	17	27	41	8	17	8	14
**Attempted Suicides**	1195	509	622	452	2202	1823	701	671
**– Male**	511	218	291	186	1049	872	224	184
**- Female**	684	291	331	266	1153	951	477	487
**Lethality**	15.07	10.54	18.48	21.80	2.87	3.80	5.65	6.81
**- Male**	22.46	16.48	28.15	31.37	5.15	5.93	13.18	15.98
**- Female**	8.56	5.52	7.54	13.36	0.69	1.76	1.65	2.79

### Overall gender differences in the distribution of completed and attempted suicides and lethality

The final data set comprised 8,942 suicidal acts: 767 completed suicides (8.6%) and 8,175 attempted suicides (91.4%). 74.4% (571 of 767 cases) of suicides and 43.2% (3,535 of 8,175 cases) of suicide attempts were performed by men.

Overall, the suicide rate in all eight studied regions (893,523 males, 958,061 females on average per year) was 21.3/100,000 for males and 6.8/100,000 for females, resulting in a male: female ratio of 3.1:1. The overall lethality was significantly higher for men (13.9%) than for women (4.1%) (phi = 0.18; χ^2^ = 274.94; df = 1; p < 0.00001) (male: female ratio of lethality: 3.43).

### Gender differences in the choice of method and the method-specific lethality

Men and women significantly differed overall in the choice of methods for suicidal acts (χ^2^ = 603.95; df = 8; p < 0.000001), due to a male preponderance for all suicide methods except “self-poisoning by psychotropic drugs”.

The overall lethality of suicidal acts for different methods varied from 1.8% (sharp objects) to 60.0% (firearms). For all methods except drowning the lethality in men exceeded that in women (see Table 2) with these differences being significant for hanging (53.0 versus 30.4%; p < 0.001), sharp objects (2.7 versus 0.9%; p = 0.009), moving objects (31.8 versus 10.0%; p = 0.002), jumping (36.7 versus 25.5%; p = 0.03), and poisoning by other means than drugs (14.1 versus 5.9%; p = 0.02).

**Table 2:** Gender differences in method-specific lethality.

Suicide method^a^	Lethality (in %)	Lethality (ratio completed suicides/suicidal acts)			
		Men	Women	φ	χ^2^ test (p)
**Poisoning by drugs (X60 –X64) (N = 5173)**	1.8	2.2 (42/1876)	1.5 (51/3297)	0.03	3.24 (p = 0.07)
**Poisoning by other means (X65 –X69) (N = 321)**	10.6	14.1 (26/185)	5.9 (8/136)	0.13	5.53 (p = 0.02)
**Hanging (X70) (N = 845)**	47.5	53.0 (338/638)	30.4 (63/207)	0.19	31.85 (p<0.0001)
**Drowning (X71) (N = 264)**	8.3	7.6 (12/157)	9.3 (10/107)	-0.03	0.24 (p = 0.62)
**Firearms (X72 –X75) (N = 45)**	60.0	63.2 (24/38)	42.9 (3/7)	0.15	p = 0.41^b^
**Sharp object (X78) (N = 1597)**	1.8	2.7 (22/827)	0.9 (7/770)	0.07	6.86 (p = 0.009)
**Jumping (X80) (N = 326)**	31.6	36.7 (65/177)	25.5 (38/149)	0.12	4.71 (p = 0.03)
**Moving object (X81,X82) (N = 148)**	23.0	31.8 (28/88)	10.0 (6/60)	0.25	9.60 (p = 0.002)
**Other methods (X76, X77, X79, X83, X84) (N = 223)**	10.8	11.7 (14/120)	9.7 (10/103)	0.03	0.22 (p = 0.64)

**Notes:** The two-sided χ ^2^ tests refer to two-by-two tables (gender (male/female) x suicide (yes/no)). φ: phi coefficient.

^a^ Definition according to ICD-10 [20].

^b^ based on two-sided exact tests by Fisher.

According to the median split for lethality (10.8%), drowning, sharp objects, poisoning by drugs and other means as well as ‘other suicide methods’ were identified as low-risk methods. Firearms, hanging, jumping and moving objects form the group of high-risk methods. Whereas 69.0% of the high-risk suicidal acts (941 of 1364) were engaged in by men, this was only the case for 41.8% of low-risk suicidal acts (3165 of 7578) (χ^2^ = 344.95; df = 1; p < 0.000001).

### Choice of suicide methods and method-specific lethality as possible determinants of gender differences in the lethality of suicidal acts

In the hypothetical case that women would choose the same methods than men, the lethality would still remain higher in men (13.91 versus 8.23) because of the higher method specific lethality of suicidal acts for men (see S1 Table).

A Poisson regression analysis was applied in order to identify relevant predictors for gender differences regarding the lethality of suicidal acts. It revealed that they were significantly associated with gender differences for the choice of methods and for method-specific lethality of suicidal acts (p < 0.00001). The contribution of gender differences for the choice of suicide methods was (non-significantly) stronger (OR = 2.03; 95% CI = 1.65 to 2.50; p < 0.000001) than that of gender differences for method-specific lethality of suicidal acts (OR = 1.64; 95% CI = 1.32 to 2.02; p = 0.000005). The interaction of the two factors was not significant (OR = 1.03; 95% CI = 0.79 to 1.35; p = 0.82).

### Predictors of the lethality of suicidal acts

According to a multivariate binary logistic regression analysis the variables country, gender, and method of suicidal acts explained 48.8% of variance in the lethality of suicidal acts (Nagelkerke’s R^2^ = 0.49; p < 0.001). Country and the method of suicidal acts could be identified as statistically significant predictors for the lethality (see Table 3). There was a statistical tendency for significantly elevated lethality of suicidal acts in men (OR = 1.60; 95% CI = 0.93 to 2.75; p = 0.09). In this model the lethality in Germany (13.8%) was found to be significantly lower than that in Hungary (19.9%; odds ratio (OR) = 3.15; 95% CI = 2.05 to 4.85; p < 0.001), but significantly higher than that in Ireland (OR = 0.14; 95% CI = 0.08 to 0.23; p < 0.001) and Portugal (OR = 0.49; 95% CI = 0.29 to 0.84; p = 0.009).

**Table 3:** Predictors of lethality following the results of a multivariate binomial logistic regression analysis for all four countries.

Independent variables	P	OR (95% CI)
**Gender Male gender (ref = female)**	0.09	1.60 (0.93; 2.75)
**Country (ref = Germany)**	<0.001	—-
**- Hungary**	<0.001	3.15 (2.05; 4.85)
**- Ireland**	<0.001	0.14 (0.08; 0.23)
**- Portugal**	0.009	0.49 (0.29; 0.84)
**Method of suicidal acts (ref = poisoning by drugs)**	<0.001	—-
**- Poisoning by other means**	<0.001	4.32 (1.95; 9.54)
**- Hanging**	<0.001	54.71 (33.31; 89.86)
**- Drowning**	<0.001	17.52 (7.95; 38.62)
**- Firearms**	<0.001	101.72 (20.02; 516.90)
**- Sharp objects**	0.59	0.80 (0.36; 1.80)
**- Jumping**	<0.001	21.82 (13.12; 36.30)
**- Moving objects**	<0.001	8.48 (3.34; 21.49)
**- Other suicide methods**	<0.001	5.13 (2.45; 10.74)
**Country x gender (ref = Germany x gender)**	0.04	—-
**- Hungary x gender**	0.23	0.72 (0.42; 1.23)
**- Ireland x gender**	0.25	1.43 (0.78; 2.62)
**- Portugal x gender**	0.10	1.75 (0.91; 3.38)
**Method of suicidal acts x gender**	0.47	—-

**Notes:** CI: confidence interval; OR = odds ratio; ref = reference category.

Compared to poisoning with drugs, all other methods (except for sharp objects) showed a significantly higher lethality (OR > = 4.32; p < 0.001). However, gender differences in lethality were not modulated by the factor “method of suicidal acts” (see Table 3). The significant interaction of the factors “country” and “gender” in this model (p = 0.04) was due to the fact that the male: female ratio for the lethality of suicidal acts was, by trend, higher in Portugal (6.59; lethality in men: 14.5%; lethality in women: 2.2%) than in Germany (2.70; lethality in men: 20.8%; lethality in women: 7.7%) (OR = 1.75; 95% CI = 0.91 to 3.38; p = 0.095).

### Additional analyses

For sensitivity analyses see S1 Text and S2 and S3 Tables.

**Fig 2:**
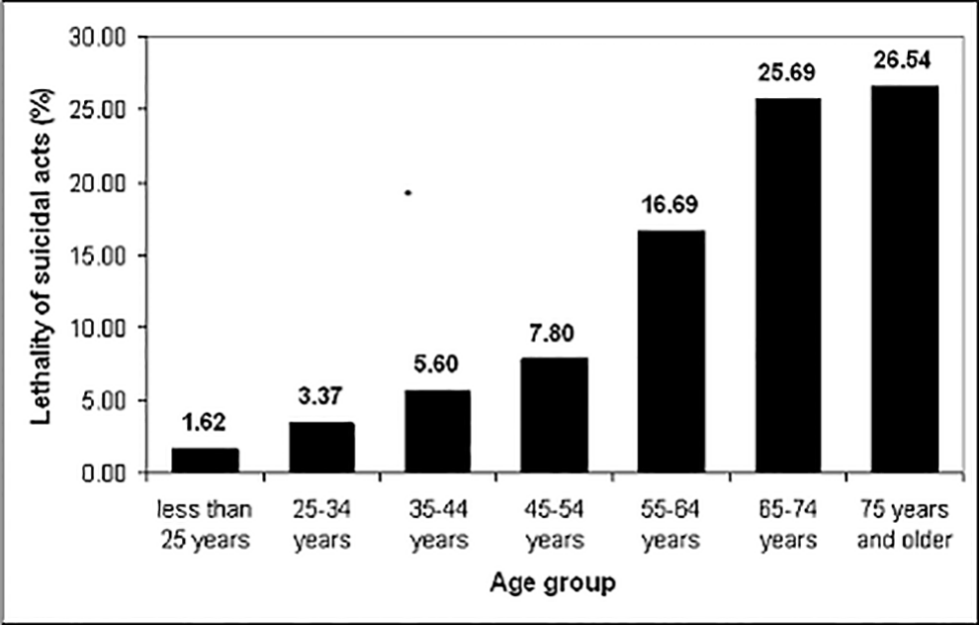
Lethality of suicidal acts in seven age groups.

The analyses concerning the variable age were restricted to Germany, Hungary, and Portugal since information about age was not available for suicides in Ireland. In comparison to young people (age less than 25 years; lethality: 1.6%) older persons were characterized by significantly higher lethality (continuously increasing from 3.4% (25–34 years) to 26.5% (75 years and older) (see Fig 2).

However, the factor age did not contribute to the gender differences in lethality. The overall median split for age was determined at 35–44 years and did not differ according to gender, neither for the whole dataset, nor for most of the single methods. The median age for the method of drowning was lower for both genders at 25–34 years. The only gender differences present were for the suicide methods “hanging” and the use of firearms: Regarding hanging, the median age for men was 35–44 years, for women 25–34 years (Z = -2.90; p = 0.004). Regarding firearms, the median age for men was 55–64 years, and for women was 45–54 years. These numbers referred to 42 cases of suicides only, thus the lack of significant age differences (Z = -0.88; p = 0.38) might primarily reflect the low number of cases. The regression analysis demonstrated that gender differences in lethality were not modulated by age: The corresponding twofold interaction failed to be significant (see Table 4).

**Table 4:** Predictors of lethality following the results of a multivariate binomial logistic regression analysis for three countries (Germany, Hungary, Portugal).

			95% CI OR	
Independent variables	p	OR	Lower limit	Upper limit
**Gender (ref = female)**	0.02	3.65	1.25	10.66
**Age (ref = 24 years and younger)**	<0.000001	—-	—-	—-
**- 25–34 years**	0.008	3.73	1.40	9.96
**- 35–44 years**	0.004	3.99	1.54	10.34
**- 45–54 years**	0.0002	5.80	2.30	14.59
**- 55–64 years**	<0.000001	12.09	4.74	30.84
**- 65–74 years**	<0.000001	16.75	6.40	43.81
**- 75 years and older**	0.00001	8.76	3.35	22.91
**Country (ref = Germany)**	<0.000001	—-	—-	—-
**- Hungary**	0.00001	2.68	1.72	4.17
**- Portugal**	0.002	0.42	0.24	0.72
**Suicide methods (ref = poisoning by drugs)**	<0.000001	—-	—-	—-
**- Poisoning by other means**	0.0004	4.39	1.93	9.98
**- Hanging**	<0.000001	40.11	22.50	71.50
**- Drowning**	0.0001	8.34	2.80	24.84
**- Firearms**	0.00005	89.88	10.33	782.26
**- Sharp objects**	0.87	1.07	0.47	2.44
**- Jumping**	<0.000001	22.45	13.01	38.74
**- Moving objects**	0.00004	7.81	2.94	20.75
**- Other suicide methods**	0.00002	5.36	2.49	11.55
**Country x gender**	0.12	—-	—-	—-
**Age x gender**	0.41	—-	—-	—-
**Suicide method x gender**	0.59	—-	—-	—-

**Notes:** CI: confidence interval; OR = odds ratio; ref = reference category. The analyses were restricted to Germany, Hungary, and Portugal since information about age was not available for suicides in Ireland.

Additional data on intentionality based on the Feuerlein scale [22] revealed that within all non-fatal suicidal acts with known intentionality, the overall proportion of serious suicide attempts with a clear motivation to die, was significantly higher in men (1,207 of 2,115 suicide attempts (57.07%)) than in women (1,508 of 3,100 suicide attempts (48.65%)) (χ^2^ = 35.74; df = 1; p < 0.000001).

Subsequent analyses for single suicide methods demonstrated that men had higher rates of serious suicide attempts than women, with hanging being an exception: For this suicide method, the corresponding rate in women was slightly higher than that in men; however, the gender difference failed to be statistically significant. Regarding suicide attempts by sharp objects, men were characterized by a significantly higher rate of serious suicide attempts, as compared to women (49.3% versus 39.6%; p = 0.03) (see S4 Table). The same was true for poisoning by drugs (53.5% versus 47.7%; p = 0.001).

## Discussion

The present study analysed several factors potentially contributing to gender differences in the lethality of suicidal acts. It generated new outcomes contributing to the scientific discussion in the field, as well as providing further support for previous findings.

### Gender differences in the choice of method

The present study conducted in four European countries revealed consistent major gender differences in the choice of suicide and attempted suicide methods. Men had a greater risk of choosing high-risk methods with significantly higher lethality than women. Compared to women, they used hanging more often for completed suicide and sharp objects more often for attempted suicide. Women used drug poisoning more often for both attempted and completed suicide than men.

These findings are in line with the outcomes of previous studies.

An Australian study [5] demonstrated significantly higher lethality of suicidal acts in men and older people for different suicide method, excluding lying in front of a moving object and jumping. A Taiwanese study [25] characterized males as having a significantly higher lethality of suicidal acts than women (26.3% versus 10.2%). Significant differences between males and females in the lethality of suicidal acts (males > females) were also found in a population based study from Illinois [26] for all suicide methods (except firearms). Similarly, a second US study [27] demonstrated higher lethality of suicidal acts in men as compared to women after stratification by suicide method. A third US study [28] confirmed consistently higher method-specific lethality of suicidal acts in men, compared to women.

As discussed, several reasons for gender differences have been put forth previously. Womens’ preference for less violent methods may be influenced by the lower level of knowledge and technical skills needed for the methods they choose [3,15] or their wish that the body and face are not severely injured [4,15]. Men’s preference for high-risk methods may be influenced by their need to not “fail”, even in suicide [4], so that they have to demonstrate success, power and restricted emotionality [10]. A Hungarian study conducted in the context of the OSPI-Europe project during the intervention period supports these findings. It was carried out independently from the retrospective recording of the suicide attempts by one of the coauthors (MT) and consisted in interviews with suicide attempters right after their detoxification in the hospital. Of 50 men and 92 women asked if they were dissapointed about the outcome of their suicide attempt, men stated significantly more often than women that this was the case (22.00% versus 7.61%; χ^2^ = 6.06; df = 1; p = 0.014) [29].

### Gender differences in the method-specific lethality and their predictors

Not only was the overall lethality significantly higher in men, but also the method-specific lethality for the methods hanging, jumping, moving objects, sharp objects, and poisoning by other means. That gender differences for the lethality of suicidal acts failed to be significant in the case of firearms (63% in males, only 43% in females) was mainly due to the small number of cases (N = 45). It should be noted that use of guns as suicide method is far less common in Europe, as compared to the United States (US) (e.g., [30]). In the US, suicidal acts involving guns are lethal in over 95% of the cases (as opposed to 60.0% in our European sample) [26]. Possibly, tampering with firearms without fire off is more frequently classified as suicidal act in Europe than in America. The calibre of the weapon used might be associated with the lower European lethality rate using guns, as well [31]. Moreover, US citizens are often more familiar with guns and other weapons in comparison to Europeans [32]. The use of weapons as suicide method in Europe is often mainly found in professions which use weapons and are characterized by male preponderance (soldiers, police forces, hunters) [33]. Women are often not so familiar with this kind of suicide method and use it in a false way (e.g., by shooting into the temple, resulting in blindness rather than death) (personal communication by Arnim Schmidtke).

Considering these findings, it was appropriate to analyse the extent to which these lethality differences differ between countries with different cultures and/or can be explained by age. Regression analysis with gender, age, country and suicide method as independent variables revealed non-significant interaction of the factors “gender” and “country” regarding the lethality of suicidal acts. The same was true for the interaction of the factors “gender” and “age”. Thus, neither context in specific European countries nor age could explain the identified gender differences in lethality of suicidal behaviour. These differences consistently present regardless of there being marked cultural differences in rates of completed suicides [1], as well as significant differences in the overall lethality rates across the four countries. Overall, lethality was higher in Hungary and Germany. However, this finding should be treated with caution because it may reflect differences in the completeness with which attempted suicides have been assessed as well as differences in suicide registration.

Age was also found to be a relevant predictor for lethality in addition to male gender and method of suicidal act. Lethality increased with age, but higher lethality in men was not explained by older age at time of the suicidal act, which is in line with the findings by Cibis et al. [3].

In this context, a comparison of the age-by-sex pattern of suicidal acts in our study with the corresponding patterns in non-European countries would have been interesting, but would have clearly exceeded the limits of our study. For example, the rather low suicide rate among older women (>64 years) in the OSPI sample (as compared to many non-European countries) might primarily reflect the fact that three of the four countries participating in the OSPI project (Germany, Ireland and Portugal) were high-income countries [1]. This finding is relevant because older women in low- and middle-income countries have been shown to reveal much higher suicide rates than older women in high-income countries [1].

### Choice of suicide methods and method-specific lethality as possible determinants of gender differences in the lethality of suicidal acts

Differences in the choice of suicide methods (OR = 2.03) more strongly contributed to gender differences in the lethality of suicidal acts than differences in the method-specific lethality of suicidal acts (OR = 1.64). Hence, it can be seen as a robust finding that men overally choose their suicide method differently than women. Such factors as the technical availability and the availability of knowledge on means of suicidal acts, personality aspects (e.g. impulsivity, aggressivity) or preference for esthetic reasons might help to determine this. At the same time, even though to a less extent contributing to the explanation of gender differences in suicide lethality, men conduct a suicidal act differently than women independent from the method chosen. While factors such as the communicative and supportive context [3,9,10] of the suicidal act influencing the chance of receiving help in time after a suicide attempt have been discussed earlier in this regard, our study explored the potential influence of intentionality, which might moderate the method specific lethality and seems to be stronger for men than women [34,35]. Indeed, suicide attempts by males had been rated as being more serious independent from suicide methods used (with the exception of hanging) suggesting gender differences in intentionality associated with suicidal behaviour.

### Strengths and weaknesses of the present study

The strengths of this study include the contribution of data from several European countries, enabling the examination of gender differences in an international setting.

Furthermore, the calculation of the influence on gender differences in lethality simultaneously by both choice of method and method-specific lethality enables a systematic approach in determining the amount of the influence of each of the individual factors.

Despite these novel approaches to the study of gender differences in the lethality of suicidal acts, the study also faced some limitations. Due to restrictive data security regulations to ensure anonymity in Ireland, specific ages could not be provided because of the relatively low absolute numbers of suicide in the intervention and control regions. Therefore analyses of the interaction between gender and age could only be conducted for three of the four countries. Attempted suicides were assessed for patients presenting to emergency departments or treated in hospitals. An unknown rate of attempted suicides remained undetected, for example cases that were presented to practices of general practitioners only. This may have caused an overestimation of the lethality of certain methods. This effect can be expected to be larger for intentional overdoses of drugs than for more lethal suicide methods requiring specialized care following survival. Moreover, the detection of attempted suicides and the registration of completed suicides might have differed across the four countries. Some suicides might be hidden and misclassified as undetermined deaths [36,37], suggesting to conduct a sensitivity analysis including undetermined deaths. This was additionally carried out within this study. However, when lethality was measured by taking undetermined deaths into account, the main results (“age”, “gender” and “country” as significant predictors for the lethality of suicidal acts) remained unchanged. Therefore the analysis and results are outlined in the supplemental material to the manuscript only. Our efforts to obtain a more complete assessment of attempted suicides compared to previous studies [3,24] may also have led to the relatively low level of lethality in this study.

### Implications for future research & policy making

Consistent with previous research into the same topic, the present study has shown remarkable gender differences in the lethality of suicidal behaviour. To our knowledge, this is the first time that the importance of two of the widely discussed contributing factors could be demonstrated in a cross-national setting: In all countries, males consistently prefer more lethal methods and present a higher method-specific lethality. This is remarkable as it holds across overall country differences in suicide rates and registrated rates of suicide attempts. The differences in the overall cross-national lethality are beyond the level of chance. This indicates that individual gender specific factors that influence suicidal acts are not majorly influenced by contextual issues such as differences in national health care system structures. Hence, efforts to develop EU-wide gender-specific guidelines or, in a latter step, prevention strategies targeting suicidal behaviour seem justified and promising.

EU gender specific suicide prevention guidelines and strategies are needed, but there are still too many gaps in the state of knowledge to present clear strategies. More research is needed to strengthen the evidence base and fill in these gaps, including support for further studies in this field. One of the factors deserving attention is the establishment of unified and streamlined procedures for the definition, assessment and evaluation of suicidal behaviour. The registration of attempted suicides, for instance, is hardly given attention to in national or even cross-national contexts. The establishment of national registries of suicidal behaviour in the clinical context might be one measure to assure an ongoing assessment, given that a registration can be achieved with sufficient reliability and validity. On the other hand, more specific knowledge on factors influencing gender specific behaviour is necessary. Future studies therefore should aim for in-depth conclusions about motivational and intentional behaviour behind the visible choice of a suicide method or a more or less lethal outcome of a suicide attempt. Those factors, and especially the interplay in between the different moderating variables, cannot be explored in detail via epidemiological studies stemming from quantitative data analysis alone. Consequently, additional methodological appraisals such as qualitative interview studies with suicide attempt survivors are needed.

Nevertheless, based on the results of the present study, some initial ideas for potential cross-national policies and intervention strategies can be proposed:

Firstly, based on the finding that men do choose more lethal methods than women and seem to carry out a suicide attempt with a more serious intent to die, gender-specific awareness campaigns addressing typically male attitudes motivating this behaviour could be developed, and delivered in predominantly male environments (e.g., football and similar sports clubs). This is especially true if the reluctance of male suicide attempters to fail is explaining higher lethality. A campaign, aiming at a turn-around in this attitude towards openly seeing the ability to display skills for coping with a crisis and seeking help as measures of success, while completing suicide would be seen as a failure could potentially contribute to a beginning change of reasoning in males.

Secondly, our results suggest to more cautiously assess and proactively follow-up for individuals at risk for suicidal acts, especially for men. For instance, emergency department and / or hospital staff members could be trained to precisely explore the severity of the wish to die for suicidal patients. Such trainings could be conceptualised as easy-to-conduct short-term-sessions or webinars, including the topics of gender differences in suicide and lethality aspects, raising awareness for specific needs for both genders, as well as developing communicative skills to purposefully and successfully address them.

Furthermore, when exploring gender specific ways to reduce the occurrence of suicidal acts, attention should be given to the availability of methods. While the physical access to lethal means only seems to be reduceable for the overall population via legislation or restrictive measures, the awareness of single, specifically lethal suicide methods might be influenceable via social processes of model learning steered by the media. This could be realised via two strategies: 1) restraining from reporting about prominent suicide cases that could serve as a role model for others, especially if highly lethal methods are used and the person committing the act is male and 2) strenghtening the association between non or less lethal methods and suicide by reporting cases of survivors that have applied less lethal methods rather than those resulting in death. Both ways could contribute to a decrease of the awareness and hence knowledge of more lethal suicide methods.

Overall, the present findings enrich the discussion of the spectrum of reasons for gender differences in lethality of suicidal behaviour and development of gender specific strategies of suicide prevention.

## Supporting Information

S1 Text Sensitivity analyses. (DOC)

S2 Text Strobe checklist. (DOCX)

S1 Table Gender differences regarding lethality for different constellations regarding the choice of methods and method-specific lethality. (DOC)

S2 Table Country- and gender-specific lethality including undetermined deaths. (DOC)

S3 Table Predictors for the lethality of suicidal acts following the results of two multivariate binomial logistic regression analyses for three countries (Germany, Hungary, Portugal) with and without inclusion of undetermined deaths. (DOC)

S4 Table Gender differences in rates of serious suicide attempts. (DOC)
