# Supplementary material for: What Are Reasons for the Large Gender Differences in the Lethality of Suicidal Acts? An Epidemiological Analysis in Four European Countries
Source: PLoS One. 2015 Jul 6;10(7):e0129062. doi: 10.1371/journal.pone.0129062 (PMC4492725; doi:10.1371/journal.pone.0129062)
Supplement: S1 Text — (DOC) [file pone.0129062.s001.doc]

### Supplemental Text: Sensitivity analyses

Sensitivity analyses were conducted concerning deaths of undetermined intent. As external causes of morbidity and mortality (ICD-10 codes Y10-Y34) [20] these cases – from now on referred to as undetermined deaths - might represent probable suicides [36]. To check whether lethality changes when including undetermined deaths, the following formula was employed: (undetermined deaths + completed suicides) / (undetermined deaths + completed suicides + suicide attempts). A regression analysis was performed for Germany, Hungary and Portugal with the same variables as described for the binary logistic regression analyses in the method section above, excluding suicide methods, because for undetermined deaths no method used could be obtained.

By comparing the lethality based on completed suicides to that based on completed suicides + undeterminded deaths, no major differences were found. The only significant difference was found for the city of Leipzig in Germany (p < 0.05), where the lethality including undetermined deaths was higher compared to the lethality regarding suicidal acts only (see Supplemental Table 2). Regression analyses revealed only two marked differences between the results (see Supplemental Table 3). A significant interaction of the factors “gender” and “country” was only present if analyses had been restricted to suicidal acts (see also Table 3). In contrast, a significant interaction of the factors “gender” and “age group” in the regression model (p = 0.03) was only found if both suicidal acts and undetermined deaths were considered. This finding was due to the fact that the male : female ratio for the lethality of suicidal acts was significantly lower in the elderly (minimum age: 75 years) (3.09) than in the reference group with a maximum age of 24 years (6.60). Related to this, there was a significantly higher male : female ratio for the lethality of suicidal acts in Portugal, as compared to Germany (which was only found, if only suicidal acts were considered). Both latter findings should not be overinterpreted since the corresponding odds ratios (0.62 versus 0.33 and 2.20 versus 1.37, respectively) did not significantly differ.

*Please insert here Supplemental Tables 2 and 3.*
