# Supplementary material for: What Are Reasons for the Large Gender Differences in the Lethality of Suicidal Acts? An Epidemiological Analysis in Four European Countries
Source: PLoS One. 2015 Jul 6;10(7):e0129062. doi: 10.1371/journal.pone.0129062 (PMC4492725; doi:10.1371/journal.pone.0129062)
Supplement: S2 Text — (DOCX) [file pone.0129062.s002.docx]

STROBE Statement—checklist of items that should be included in reports of observational studies

|  | Item No | Recommendation |
| --- | --- | --- |
| **Title and abstract** | 1 | (*a*) Indicate the study’s design with a commonly used term in the title or the abstract |
|  |  | (*b*) Provide in the abstract an informative and balanced summary of what was done and what was found  ***Response:***  ***a) The present study was conducted as an epidemiological study within the context of an international prospective cohort study. This is mentioned at the beginning of the Methods section in our article. The study design has been indicated in the title, abstract (see “Design” at page 4) as well as in the last paragraph of the introduction (see page 9-10).***  ***b) An informative and balanced summary of what was done and what was found has been provided in the abstract of the present article (see pages 4-6).*** |
| Introduction | | |
| Background/rationale | 2 | Explain the scientific background and rationale for the investigation being reported  ***Response:***  ***In our Introduction, we explain that it is important to gain a better understanding of the relevance of the many factors associated with the large gender differences in lethality of suicidal acts in order to be able to improve preventive actions and gender-specific care for suicidal patients (see pages 7-9).*** |
| Objectives | 3 | State specific objectives, including any prespecified hypotheses  ***Response:***  ***We clearly state our prespecified objectives at page 9 as follows:***  ***“More specifically the study will address 1) the extent to which lethality differences are explained by gender differences in the choice of more or less lethal suicidal methods, 2) gender differences in lethality within certain suicidal methods, 3) the interaction of the two factors in predicting the extend of gender differences in lethality and 4) whether gender differences in the lethality of suicidal acts differ between countries and age groups. In addition, lethality differences in age and gender differences in the intentionality of suicide attempts were explored******.”*** |
| Methods | | |
| Study design | 4 | Present key elements of study design early in the paper  ***Response:***  ***We present key elements of the study design in the first paragraph of the Methods section at page 9-10.*** |
| Setting | 5 | Describe the setting, locations, and relevant dates, including periods of recruitment, exposure, follow-up, and data collection  ***Response:***  ***We provided this data in the first paragraph of the Methods section (see page 9-10).*** |
| Participants | 6 | (*a*) *Cohort study*—Give the eligibility criteria, and the sources and methods of selection of participants. Describe methods of follow-up  ***Response:***  ***This information is provided in the Methods section (see chapters “Assessment of completed suicides” as well as “Assessment of attempted suicides” at pages 11-12).***  *Case-control study*—Give the eligibility criteria, and the sources and methods of case ascertainment and control selection. Give the rationale for the choice of cases and controls  *Cross-sectional study*—Give the eligibility criteria, and the sources and methods of selection of participants |
|  |  | (*b*) *Cohort study*—For matched studies, give matching criteria and number of exposed and unexposed  ***Response:***  ***Not applicable.***  *Case-control study*—For matched studies, give matching criteria and the number of controls per case |
| Variables | 7 | Clearly define all outcomes, exposures, predictors, potential confounders, and effect modifiers. Give diagnostic criteria, if applicable  ***Response:***  ***We described the primary outcome (lethality of suicidal acts) in the Methods section in the chapter about “Definition of lethality, high / low risk methods and intentionality of suicidal acts” at page 13. In this chapter, the second outcome (intentionality of suicidal acts as assessed by the Feuerlein scale) is also described (see page 13). The primary variable of interest was the sex of persons committing a suicidal act (see Methods section; chapter about “Statistical analysis” at page 13-15). We listed potential predictors and confounders in this chapter, too (including age, country, suicide methods; see page14).*** |
| Data sources/ measurement | 8* | For each variable of interest, give sources of data and details of methods of assessment (measurement). Describe comparability of assessment methods if there is more than one group  ***Response:***  ***For each variable of interest, sources of data and details of methods of assessment have been given (see Methods section, especially the chapters about “Assessment of completed suicides”, “Assessment of attempted suicides” and “Definition of lethality, high / low risk methods and intentionality of suicidal acts” at pages 11-13).*** |
| Bias | 9 | Describe any efforts to address potential sources of bias  ***Response:***  ***It has been emphasized in the Methods section (see chapter about the “Assessment of attempted suicides” at page 11-12) that a standardised questionnaire for data assessment and a codebook listing the variables for the registration of attempted suicides had been applied by all partners to ensure comparability in data obtainment. Moreover, it had been assured that data assessment was consistent over time within each study centre.*** |
| Study size | 10 | Explain how the study size was arrived at  ***Response:***  ***Table 1 shows how the study size had been arrived at by the eight regions providing data for suicidal acts in predefined periods in the context of the OSPI-Europe project.*** |
| Quantitative variables | 11 | Explain how quantitative variables were handled in the analyses. If applicable, describe which groupings were chosen and why  ***Response:***  ***We described which variables were in which statistical model (see Methods section; chapter about “Statistical analysis” at pages 13-15). In this context, categorizations of quantitative variables had been specified and justified.*** |
| Statistical methods | 12 | (*a*) Describe all statistical methods, including those used to control for confounding |
|  |  | (*b*) Describe any methods used to examine subgroups and interactions |
|  |  | (*c*) Explain how missing data were addressed |
|  |  | (*d*) *Cohort study*—If applicable, explain how loss to follow-up was addressed  *Case-control study*—If applicable, explain how matching of cases and controls was addressed  *Cross-sectional study*—If applicable, describe analytical methods taking account of sampling strategy |
|  |  | (*e*) Describe any sensitivity analyses  ***Response:***  ***The chapter about “Statistical analysis” in the Methods section (see pages 13-15) clearly describes all statistical methods, including those used to control for confounding. In particular, statistical methods used to examine subgroups and interactions (e.g., regression analyses) had been described. It had been made clear that in cases of missing values (e.g., considering age) restricted data sets had been used. Loss to follow-up had not been addressed because suicidal acts had been investigated. All sensitivity analyses had been addressed and results were given in additional tables.*** |

Continued on next page

| Results | | |
| --- | --- | --- |
| Participants | 13* | (a) Report numbers of individuals at each stage of study—eg numbers potentially eligible, examined for eligibility, confirmed eligible, included in the study, completing follow-up, and analysed  ***Response:***  ***The numbers of suicidal acts committed by men versus women are reported in the first sentences of the Results section (see page 15 in the manuscript).*** |
|  |  | (b) Give reasons for non-participation at each stage  ***Response:***  ***Not applicable since the number of missing suicidal acts could not be determined.*** |
|  |  | (c) Consider use of a flow diagram  ***Response:***  ***Not necessary.*** |
| Descriptive data | 14* | (a) Give characteristics of study participants (eg demographic, clinical, social) and information on exposures and potential confounders |
|  |  | (b) Indicate number of participants with missing data for each variable of interest |
|  |  | (c) *Cohort study*—Summarise follow-up time (eg, average and total amount)  ***Response:***  ***Characteristics of the sample are given in Table 1. Number of participants with missing data for each variable of interest: Not applicable since cases of suicidal acts had been analysed. Follow-up time (“time span”) is given in Table 1.*** |
| Outcome data | 15* | *Cohort study*—Report numbers of outcome events or summary measures over time  ***Response:***  ***The number of suicidal acts is reported in the result section (1^st^ paragraph page 15) and Table 1.*** |
|  |  | *Case-control study—*Report numbers in each exposure category, or summary measures of exposure |
|  |  | *Cross-sectional study—*Report numbers of outcome events or summary measures |
| Main results | 16 | (*a*) Give unadjusted estimates and, if applicable, confounder-adjusted estimates and their precision (eg, 95% confidence interval). Make clear which confounders were adjusted for and why they were included  ***Response:***  ***Odds ratios and the corresponding 95% confidence intervals had been given (see Results section ( pages 16-17) and the corresponding tables). Possible confounders were addressed in this context (see comment 7 and the Results section regarding regression analyses).*** |
|  |  | (*b*) Report category boundaries when continuous variables were categorized  ***Response:***  ***Done.*** |
|  |  | (*c*) If relevant, consider translating estimates of relative risk into absolute risk for a meaningful time period  ***Response:***  ***Not relevant.*** |
| Other analyses | 17 | Report other analyses done—eg analyses of subgroups and interactions, and sensitivity analyses  ***Response:***  ***This was done (see in particular the supplemental materials).*** |
| Discussion | | |
| Key results | 18 | Summarise key results with reference to study objectives  ***Response:***  ***The study addressed the question to which extent gender differences in the lethality of suicidal acts were explained by gender differences in the choice of more or less lethal suicidal methods and gender differences in lethality within certain suicidal methods.***  ***It could be demonstrated that higher method-specific lethality of suicidal acts by males clearly contributes to the higher overall lethality of suicidal acts in males although to a lesser degree than the choice of more lethal methods by males.***  ***Moreover, it was investigated whether gender differences in the lethality of suicidal acts differed between countries and were explainable by age.***  ***Gender differences in the lethality of suicidal acts were found to be widely consistent across four European countries (Germany, Hungary, Ireland, Portugal) and independent from age differences.***  ***In addition, gender differences in the intentionality of suicide attempts were explored.***  ***Suicide attempts by males were rated as more serious independent of the method used (with the exception of hanging) suggesting gender differences in intentionality associated with suicidal behavior.*** |
| Limitations | 19 | Discuss limitations of the study, taking into account sources of potential bias or imprecision. Discuss both direction and magnitude of any potential bias  ***Response:***  ***The limitations of the study had been addressed as follows (see pages 21-22):***  ***“Due to restrictive data security regulations to ensure anonymity in Ireland, specific ages could not be provided because of the relatively low absolute numbers of suicide in the intervention and control regions. Therefore analyses of the interaction between gender and age could only be conducted for three of the four countries. Attempted suicides were assessed for patients presenting to emergency departments or treated in hospitals. An unknown rate of attempted suicides remained undetected, for example cases that were presented to practices of general practitioners only as well as those that never sought professional help at all. This may have caused an overestimation of the lethality of certain methods. This effect can be expected to be larger for intentional overdoses of drugs than for more lethal suicide methods requiring specialized care following survival. Moreover, the detection of attempted suicides and the registration of completed suicides might have differed across the four countries. Some suicides might be hidden and misclassified as undetermined deaths], suggesting to conduct a sensitivity analysis including undetermined deaths. This was additionally carried out within this study. However, when lethality was measured by taking undetermined deaths into account, the main results (“age”, “gender” and “country” as significant predictors for the lethality of suicidal acts) remained unchanged. Therefore the analysis and results are outlined in the supplemental material to the manuscript only. Our efforts to obtain a more complete assessment of attempted suicides compared to previous studies may also have led to the relatively low level of lethality in this study. It can be stated that current estimations of method-specific lethality need to be reconsidered and that a reliable calculation of method-specific lethality requires as complete an assessment of attempted suicides as possible.”*** |
| Interpretation | 20 | Give a cautious overall interpretation of results considering objectives, limitations, multiplicity of analyses, results from similar studies, and other relevant evidence  ***Response:***  ***We attempted to do so in the Discussion section (see pages 19-24).*** |
| Generalisability | 21 | Discuss the generalisability (external validity) of the study results  ***Response:***  ***We emphasized in the Discussion section that the strengths of this study include the contribution of data from several European countries, enabling the examination of gender differences in an international setting (see page 21).*** |
| Other information | | |
| Funding | 22 | Give the source of funding and the role of the funders for the present study and, if applicable, for the original study on which the present article is based  ***Response:***  ***We provided this information in the “Funding” chapter at page 25.*** |

*Give information separately for cases and controls in case-control studies and, if applicable, for exposed and unexposed groups in cohort and cross-sectional studies.

**Note:** An Explanation and Elaboration article discusses each checklist item and gives methodological background and published examples of transparent reporting. The STROBE checklist is best used in conjunction with this article (freely available on the Web sites of PLoS Medicine at http://www.plosmedicine.org/, Annals of Internal Medicine at http://www.annals.org/, and Epidemiology at http://www.epidem.com/). Information on the STROBE Initiative is available at www.strobe-statement.org.
