# Supplementary material for: What Are Reasons for the Large Gender Differences in the Lethality of Suicidal Acts? An Epidemiological Analysis in Four European Countries
Source: PLoS One. 2015 Jul 6;10(7):e0129062. doi: 10.1371/journal.pone.0129062 (PMC4492725; doi:10.1371/journal.pone.0129062)
Supplement: S1 Table — (DOC) [file pone.0129062.s003.doc]

Supplemental Table 1: Gender differences regarding lethality for different constellations regarding the choice of methods and method-specific lethality

| **Gender differences** | | **Lethality of suicidal acts** | | |
| --- | --- | --- | --- | --- |
| **Choice of suicide methods** | **Method-specific lethality** | **Women** | **Men** | **Difference between men and women** |
| **No** | **No** | 13.92  (673/4836*100) | 13.91  (571/4106*100) | **-0.01** |
| **No** | **Yes** | 8.23  (398/4836*100) | 13.91  (571/4106*100) | **5.68** |
| **Yes** | **No** | 6.64  (321/4836*100) | 13.91  (571/4106*100) | **7.27** |
| **Yes** | **Yes** | 4.05  (196/4836*100) | 13.91  (571/4106*100) | **9.86** |
