# Supplementary material for: What Are Reasons for the Large Gender Differences in the Lethality of Suicidal Acts? An Epidemiological Analysis in Four European Countries
Source: PLoS One. 2015 Jul 6;10(7):e0129062. doi: 10.1371/journal.pone.0129062 (PMC4492725; doi:10.1371/journal.pone.0129062)
Supplement: S2 Table — (DOC) [file pone.0129062.s004.doc]

Supplemental Table 2: Country- and gender-specific lethality including undetermined deaths

| **Country** | **Germany** | | | **Hungary** | | **Ireland** | | **Portugal** | | | |
| --- | --- | --- | --- | --- | --- | --- | --- | --- | --- | --- | --- |
| **Timespan** | Jun 2008 –  May 2011 | | | Jan 2008 –  Dec 2010 | | Apr 2009 –  Mar 2011 | | Apr 2009 –  Mar 2011 | | | |
| **City** | **Leipzig** | | **Magdeburg** | **Miskolc** | **Szeged** | **Limerick** | **Galway** | **Amadora** | | **Almada** | |
| **Lethality (95%CI)** | 15.07 (13.24-17.05) | 10.54 (8.14-13.36) | | 18.48 (15.79-21.42) | 21.80 (18.50-25.39) | 2.87 (2.22-3.64) | 3.80 (2.98-4.76) | | 5.65 (4.10-7.56) | | 6.81 (5.08-8.90) |
| **- Male (95% CI)** | 22.46 (19.33-25.84) | 16.48 (12.19-21.54) | | 28.15 (23.82-32.80) | 31.37 (25.89-37.26) | 5.15 (3.93-6.63) | 5.93 (4.50-7.65) | | 13.18 (9.30-17.93) | | 15.98 (11.39-21.52) |
| **- Female (95% CI)** | 8.56 (6.65-10.79) | 5.52 (3.25-8.69) | | 7.54 (5.03-10.78) | 13.36 (9.76-17.68) | 0.69 (0.30-1.35) | 1.76 (1.03-2.80) | | 1.65 (0.71-3.22) | | 2.79 (1.54-4.64) |
| **Lethality incl. undetermined deaths (95% CI)** | 19.47 (17.49-21.58)* | 12.09 (9.55-15.03) | | 19.74 (16.99-22.72) | 23.00 (19.65-26.62) | 3.97 (3.21-4.85) | 4.25 (3.39-5.26) | | 8.84 (6.93-11.08) | | 10.89 (8.76-13.34) |
| **- Male (95% CI)** | 26.79 (23.54-30.24) | 18.35 (13.90-23.53) | | 29.88 (25.51-34.54) | 32.36 (26.87-38.24) | 6.26 (4.91-7.84) | 6.44 (4.95-8.21) | | 16.42 (12.19-21.41) | | 21.37 (16.30-27.18) |
| **-Female (95% CI)** | 12.98 (10.71-15.53) | 6.73 (4.21-10.10) | | 8.06 (5.46-11.36) | 14.74 (11.00-19.17) | 1.79 (1.11-2.72) | 2.16 (1.34-3.28) | | 4.79 (3.09-7.04) | | 6.17 (4.26-8.59) |

**Notes:** CI = confidence interval; * Significant difference between lethality and lethality including undetermined deaths (p < 0.05).
