# Supplementary material for: What Are Reasons for the Large Gender Differences in the Lethality of Suicidal Acts? An Epidemiological Analysis in Four European Countries
Source: PLoS One. 2015 Jul 6;10(7):e0129062. doi: 10.1371/journal.pone.0129062 (PMC4492725; doi:10.1371/journal.pone.0129062)
Supplement: S3 Table — (DOC) [file pone.0129062.s005.doc]

**Supplemental Table 3**: Predictors for the lethality of suicidal acts following the results of two multivariate binomial logistic regression analyses for three countries (Germany, Hungary, Portugal) with and without inclusion of undetermined deaths

| **Independent variables** | **Suicidal acts** | | **Suicidal acts + undetermined deaths** | |
| --- | --- | --- | --- | --- |
|  | **p** | **OR (95% CI)** | **p** | **OR (95% CI)** |
| **Gender (ref = female)** | 0.000057 | 6.51 (2.61; 16.20) | 0.00002 | 5.00 (2.39; 10.45) |
| **Age (ref = 24 years and younger)** | <0.000001 | --- | <0.000001 | --- |
| **- 25-34 years** | 0.009 | 3.54 (1.38; 9.08) | 0.04 | 2.29 (1.05; 4.99) |
| **- 35-44 years** | 0.002 | 4.10 (1.64; 10.25) | 0.005 | 2.88 (1.38; 6.04) |
| **- 45-54 years** | 0.000054 | 6.14 (2.54; 14.80) | 0.0002 | 3.95 (1.94; 8.04) |
| **- 55-64 years** | <0.000001 | 12.88 (5.32; 31.17) | <0.000001 | 8.81 (4.33; 17.93) |
| **- 65-74 years** | <0.000001 | 16.83 (6.83; 41.49) | <0.000001 | 11.14 (5.36; 23.16) |
| **- 75 years and older** | <0.000001 | 13.68 (5.55; 33.71) | <0.000001 | 27.33 (13.85; 53.95) |
| **Country (ref = Germany)** | <0.000001 | --- | 0.00001 | --- |
| **- Hungary** | 0.09 | 1.35 (0.95; 1.92) | 0.46 | 1.13 (0.82; 1.57) |
| **- Portugal** | <0.000001 | 0.27 (0.17; 0.45) | 0.00005 | 0.49 (0.35; 0.69) |
| **Country x gender (ref = Germany x gender)** | 0.03 | --- | 0.22 | --- |
| **- Hungary x gender** | 0.26 | 1.28 (0.84; 1.96) | 0.14 | 1.36 (0.91; 2.03) |
| **- Portugal x gender** | 0.008 | 2.20 (1.23; 3.92) | 0.16 | 1.37 (0.88; 2.14) |
| **Age x gender (ref = 24 years and younger x gender)** | 0.40 | --- | 0.03 | --- |
| **- 25-34 years x gender** | 0.05 | 0.34 (0.12; 1.00) | 0.18 | 0.54 (0.22; 1.33) |
| **- 35-44 years x gender** | 0.27 | 0.57 (0.20; 1.57) | 0.45 | 0.72 (0.31; 1.69) |
| **- 45-54 years x gender** | 0.11 | 0.44 (0.17; 1.19) | 0.38 | 0.69 (0.30; 1.58) |
| **- 55-64 years x gender** | 0.08 | 0.41 (0.15; 1.11) | 0.19 | 0.57 (0.25; 1.32) |
| **- 65-74 years x gender** | 0.31 | 0.59 (0.21; 1.65) | 0.67 | 0.83 (0.35; 1.98) |
| **- 75 years and older x gender** | 0.36 | 0.62 (0.22; 1.73) | 0.01 | 0.33 (0.14; 0.74) |

**Notes:** CI: confidence interval; OR = odds ratio; ref = reference category. The analyses were restricted to Germany, Hungary, and Portugal since information about age was not available for suicides in Ireland.
