# Supplementary material for: What Are Reasons for the Large Gender Differences in the Lethality of Suicidal Acts? An Epidemiological Analysis in Four European Countries
Source: PLoS One. 2015 Jul 6;10(7):e0129062. doi: 10.1371/journal.pone.0129062 (PMC4492725; doi:10.1371/journal.pone.0129062)
Supplement: S4 Table — (DOC) [file pone.0129062.s006.doc]

Supplemental Table 4: Gender differences in rates of serious suicide attempts

| **Suicide method^1^** | **Rate of serious suicide attempts (ratio serious suicide attempts/all suicide attempts with known intentionality*)** | | |
| --- | --- | --- | --- |
|  | **Men** | **Women** | **χ^2^ test**  **p** |
| **Poisoning by drugs (X60 – X64)** | 53.5  (651/1216) | 47.7  (1112/2329) | 10.72  (p=0.001) |
| **Poisoning by other means (X65 – X69)** | 62.0  (129/208) | 56.7  (106/187) | 1.16  (p=0.28) |
| **Hanging (X70)** | 80.3  (102/127) | 83.3  (45/54) | 0.23  (p=0.63) |
| **Drowning (X71)** | 92.3  (36/39) | 87.9  (29/33) | p=0.70^2^ |
| **Firearms (X72 – X75)** | 100.0%  (11/11) | 50.0%  (1/2) | p=0.15^2^ |
| **Sharp object (X78)** | 49.3  (134/272) | 39.6  (101/255) | 4.97  (p=0.03) |
| **Jumping (X80)** | 73.1  (49/67) | 67.1  (47/70) | 0.59  (p=0.44) |
| **Moving object (X81,X82)** | 80.8  (21/26) | 58.1  (18/31) | 3.37  (p=0.07) |
| **Other methods (X76, X77, X79, X83, X84)** | 46.2  (30/65) | 36.8  (21/57) | 1.08  (p=0.30) |

Notes: The two-sided χ^2^ tests refer to two-by-two tables (gender (male/female) x serious suicide attempt (yes/no)).

^1^ Definition according to ICD-10 [20]. ^2^ based on two-sided exact tests by Fisher.

* according to the Feuerlein scale [22].
